# Melatonin in Prevention of the Sequence from Reflux Esophagitis to Barrett’s Esophagus and Esophageal Adenocarcinoma: Experimental and Clinical Perspectives

**DOI:** 10.3390/ijms19072033

**Published:** 2018-07-13

**Authors:** Jolanta Majka, Mateusz Wierdak, Iwona Brzozowska, Marcin Magierowski, Aleksandra Szlachcic, Dagmara Wojcik, Slawomir Kwiecien, Katarzyna Magierowska, Jacek Zagajewski, Tomasz Brzozowski

**Affiliations:** 1 Department of Physiology, Faculty of Medicine, Jagiellonian University Medical College, 31-531 Cracow, Poland; jolmaj@poczta.fm (J.M.); mateusz.wierdak@uj.edu.pl (M.W.); m.magierowski@uj.edu.pl (M.M.); alsz@poczta.fm (A.S.); dagmarawojcik@interia.pl (D.W.); skwiecien@cm-uj.krakow.pl (S.K.); k.jasnos@interia.pl (K.M.); 2 Department of Anatomy, Faculty of Medicine, Jagiellonian University Medical College, 33-332 Cracow, Poland; i.brzozowska@uj.edu.pl; 3 Department of Biochemistry, Faculty of Medicine, Jagiellonian University Medical College, 31-034 Cracow, Poland; jackzag@yahoo.com

**Keywords:** melatonin, L-tryptophan, reflux esophagitis, gastroesophageal reflux disease, Barrett’s esophagus, inflammation, esophageal blood flow, esophageal adenocarcinoma

## Abstract

Melatonin is a tryptophan-derived molecule with pleiotropic activities which is produced in all living organisms. This “sleep” hormone is a free radical scavenger, which activates several anti-oxidative enzymes and mechanisms. Melatonin, a highly lipophilic hormone, can reach body target cells rapidly, acting as the circadian signal to alter numerous physiological functions in the body. This indoleamine can protect the organs against a variety of damaging agents via multiple signaling. This review focused on the role played by melatonin in the mechanism of esophagoprotection, starting with its short-term protection against acute reflux esophagitis and then investigating the long-term prevention of chronic inflammation that leads to gastroesophageal reflux disease (GERD) and Barrett’s esophagus. Since both of these condition are also identified as major risk factors for esophageal carcinoma, we provide some experimental and clinical evidence that supplementation therapy with melatonin could be useful in esophageal injury by protecting various animal models and patients with GERD from erosions, Barrett’s esophagus and neoplasia. The physiological aspects of the synthesis and release of this indoleamine in the gut, including its release into portal circulation and liver uptake is examined. The beneficial influence of melatonin in preventing esophageal injury from acid-pepsin and acid-pepsin-bile exposure in animals as well as the usefulness of melatonin and its precursor, L-tryptophan in prophylactic and supplementary therapy against esophageal disorders in humans, are also discussed.

## 1. Anti-Reflux Esophageal Barrier and Pathogenesis of Esophageal Damage Induced by Gastroduodenal Reflux

Gastroesophageal reflux disease (GERD) is a multifactorial process and one of the most common diseases of the upper gastrointestinal tract (GI-tract) in humans [1,2,3]. This disorder is the logical consequence of the failure of the physiological anti-reflux barrier to protect against frequent and abnormal load of gastroesophageal reflux of acid or alkaline content, or both. The pathogenesis of GERD is complex, resulting from an imbalance between defensive factors protecting the esophagus such as the anatomical anti-reflux barrier, esophageal acid clearance, the mucosal epithelial component of the esophageal barrier and aggressive factors from the stomach and duodenum [4,5,6]. Important aggressive endogenous factors of gastric origin, which affect esophageal mucosa during gastric reflux, predominantly include the gastric acid and the pepsin activity [7,8]. At the cellular level, the development of inflammatory alterations, known as reflux esophagitis (RE) is due to hydrogen ion diffusion into the mucosa, leading to tissue acidification and necrotic mucosal damage. It has been demonstrated that acid alone induces minor injury of esophageal mucosa at pH of less than 3. However, the acid combined with even small amounts of pepsin results in potent damage of the mucosal lining, increases the permeability of esophageal mucosa to hydrogen ions, creates morphologic changes to the esophageal mucosal structure and local hemorrhage [8,9,10]. Thus, the ability of pepsin to induce esophageal mucosa injury is acid-dependent, with maximal damage below pH 3 [5,6,7,8]. Along with acid and pepsin, the duodenal content containing bile acids, trypsin and hyper osmolality may be injurious to the esophageal mucosa during the presence of duodenogastric reflux [9,10]. Experimental studies have demonstrated that bile acids induce mucosal injury of greater magnitude when the combination of hydrochloric acid and pepsin have occurred [10]. Among aggressive factors of exogenous origin, cigarette smoking, alcohol and non-steroidal anti-inflammatory drugs (NSAIDs) such as aspirin, naproxen, indomethacin, and ibuprofen have been identified as risk factors for the development of erosive esophagitis in humans [11,12,13,14]. Development of RE is sometimes also potentiated by simultaneous steroid use. Among NSAIDs, the most popular drug, aspirin has increased the susceptibility of the esophageal mucosa to the injurious action of acid and pepsin in experimental animals and humans [15,16]. The basic level of esophageal defense against acid damage consists of natural anti-reflux mechanisms, which are created by special physiological properties of the gastroesophageal junction [4,17]. The lower esophageal sphincter (LES), is considered to play an essential role as the mechanical barrier that prevents the backflow of gastric contents from the stomach into the esophagus [6]. The accumulated evidence indicates that abnormalities in the LES, such as transient sphincter relaxation, contribute to the development of reflux of gastric content into the esophagus. The next level of esophageal luminal defense against reflux damage is primarily achieved by luminal acid clearance which is accomplished by esophageal peristalsis, removal of the esophageal contents, and neutralization of esophageal luminal acidity by bicarbonate secreted locally by submucosal glands or delivered with swallowed saliva [5,18]. The esophageal mucosal defense includes tissue resistance, which depends upon the presence of pre-epithelial, epithelial, and post-epithelial defensive mechanisms consisting of either, or both the morphological and functional components [17,18]. The morphological components include the cell layers of esophageal mucosa, which create a relatively tight epithelium with high resistance to ionic movement. The pre-epithelial functional components of mucosal resistance include mucus and unstirred water layers with bicarbonate ions located on the surface of the esophageal epithelium to neutralize and extrude hydrogen ions [5,6,19]. Functional defense components include the buffering capacity of negatively charged proteins, bicarbonate ions, and H^+^ extrusion processes [18,19]. 

The esophageal protection against the damaging effects of acidic and acid-alkaline refluxate contents depends upon the buffer capacity of the pre-epithelial and epithelial (intraepithelial) mechanisms. These mechanisms involve both the histological architecture of the mucosal membrane and the functional physiological properties of esophageal mucosa preventing the back diffusion of hydrogen ions [17,18,19]. Post-epithelial defense mechanisms provided by adequate mucosal blood flow as well as buffering capacity are detrimental in the regulation of the local acid-base balance [20,21]. In particular, the undisturbed esophageal blood flow (EBF) seems to be a critical physiological measure responsible for the proper functioning of the pre-epithelial and for the intraepithelial mechanisms ensuring the protective ability of the esophageal mucosal barrier against the damaging action of acid, pepsin and bile [21]. Moreover, adequate blood flow is responsible for the supply of oxygen and energy substitutes and the distribution of neurohormonal and humoral factors controlling the local blood flow. Studies in the past have shown that the local blood flow in the esophageal mucosa plays an essential role in maintaining optimal pH in response to mucosal exposure to gastric acid contents [20,21,22]. Indisputably, there is a close relationship between EBF, esophageal function and protective mechanisms in the esophageal mucosa. Sudden limitation or cessation of EBF may result in mucosal esophageal damage due to both hypoxia and the damaging effects of the reperfusion which follows the ischemia. The damaging effect of ischemia followed by rapid reperfusion is attributable to the enhancement in generation of highly cytotoxic reactive oxygen metabolites (ROM) [23,24]. Interestingly, the extent and depth of mucosal damage depends on the time and degree of complete cessation or reduction of EBF. The regulation of EBF depends on systemic nervous mechanisms and local hormonal-humoral factors [21,22,23,24,25]. Under physiological conditions, the EBF increases in response to increased secretory activity of the esophageal mucosa and during incidental acid reflux episodes of gastric content [23,24,25]. This local hyperemia is responsible for the bicarbonate supply which creates and maintains the alkaline pH of the mucous membrane surface and provides rapid neutralization of the acid load during reflux episodes [20,24,25]. 

The neurogenic control of esophageal integrity involves the activity of capsaicin-sensitive sensory nerves releasing vasoactive neuropeptides, which are known to ensure the protection of the esophageal epithelial barrier mucosa against damaging factors [26,27]. Esophageal sensory fibers are carried by vagal and spinal primary unmyelinated afferent nerves containing and releasing vasodilatory peptides such as calcitonin gene-related peptide (CGRP), substance P (SP) and vasoactive intestinal peptide (VIP) [26,27,28]. They are implicated in orthodromic transmission of sensory information from esophageal mucosa to the central nervous system [26,27,28]. The main physiological role of capsaicin-sensitive sensory nerves is the modulation of the esophageal mucosal hyperemia in response to physiological factors and to damaging factors due to stimulation of mucosal acid-sensing sensory endings [21,28,29]. In response to stimulation of these mucosal acid-sensing sensory endings, predominantly by the reflux of gastric acid to the esophagus, an increase in EBF has been observed [29]. Numerous vasodilatory locally released sensory neurotransmitters factors such as CGRP and VIP, as well as endothelium-derived gaseous molecules nitric oxide (NO) and hydrogen sulfide (H_2_S) were implicated in the mechanism of esophageal mucosal hyperemia [9,16,27,28]. Previous studies have strongly supported the importance of neuropeptides released from capsaicin-sensitive sensory nerve endings in the mechanism of esophageal integrity and mucosal defense [27,28,29]. Moreover, hyperemia evoked by the reflux of gastric juice in rat esophagus was inhibited by ablation of capsaicin-sensitive sensory nerves by neurotoxic dose of capsaicin, this latter effect being reversed in part by the treatment of capsaicin-sensory inactivated rats with exogenous CGRP [9,26,28].

## 2. Reflux Esophagitis in Animal Models and the Process of Progression of Mucosal Changes Caused by Reflux Esophagitis into Barrett’s Esophagus and Esophageal Adenocarcinoma

Several animal models in vivo have been developed to reproduce the mechanism of both acute and chronic inflammation of esophageal mucosa. One of these methods allows inducing acute inflammation, which has been fully described by Nagahama et al. [7]. The major rationale behind this method is from one side, the reduction of the gastric capacity by ligation of the reservoir part of the stomach, but from the other side, the pylorus ligation to prevent the passage of the gastric contents into duodenum. This acute method, which facilitates the return of gastric juice into esophagus, achieves the induction of acute hemorrhagic lesions of the esophageal mucosa in the relatively short time of a few hours [7]. Experimental in vivo methods causing chronic esophageal inflammation are strictly dependent upon the continuous contact of gastric and/or duodenal contents with esophageal mucosa. The method introduced by Tsui et al. [30] is a modification of Tsukimi and Okabe’s method of inducing chronic gastric ulceration in the stomach [31]. According to the method used by Tsui et al. [30], esophageal necrosis is initially produced by 100% acetic acid application onto an esophageal segment in a standardized area with a diameter of 3 mm for 60 s. The method created by Omura et al. [32] is based on the surgical placement of a standardized girdle in the antro-duodenal area which impedes the passage of gastric contents to the duodenum. The method published by Nishijima et al. [33] aims to create connections between the duodenum and esophagus which allow chronic passage of the gastro-duodenal contents through esophagus. The complete gastrectomy is performed with stitching of the end of duodenum and creation of the end-to-side connection between the distal esophagus and jejunum at the standardized distance from the Treitz ligament. The chronic passage of gastroduodenal contents into the esophagus can be also obtained using the surgical method [34]. The creation of the esophagoduodenal anastomosis is made by cutting the esophagus from the stomach at the level of the lower esophagus sphincter, stitching the stomach stump and then stitching the distal stump of the esophagus to the free wall of the duodenum at a distance of 1 cm from the pylorus [34]. Naito et al. [10] also developed a surgical method allowing the chronic passage of duodenal or gastro-duodenal contents through the esophagus. This method includes a 7 mm oblong cut on both the free anterior wall of the esophagus and the duodenum and performing the connections between the walls of the esophagus and duodenum using the side-to-side method. A method that allows chronic administration of test substances (drugs), that are continually infused, has also been developed. Briefly, one end of the esophageal cannula is placed in a subcutaneous pump allowing the test substance to be administered in continuous infusion at a certain rate via an operatively placed cannula into the esophagus directly into esophageal lumen [35].

Previous experimental and clinical studies have provided well documented data that RE is the main risk factor of Barrett’s esophagus (BE) and esophageal adenocarcinoma (EAC) [36,37]. The chronic irritation of esophageal mucosa caused by gastric or gastroduodenal contents are the major risk factors in the pathogenesis of these diseases. It is still unclear why some patients with GERD suffer only from RE, while others develop metaplastic and dysplastic esophageal changes [36,37,38]. BE is a condition based on a histologically proven change of esophageal epithelium to the specialized intestinal-type glandular epithelium [39,40]. The current definition of BE is defined by the endoscopic and histological criteria because diagnosis of BE requires validation by histological analysis of endoscopically obtained biopsy specimens [38]. It is well documented that BE patients are at higher risk of progression to esophageal dysplasia and EAC [39,40,41]. It is estimated that BE metaplasia increases the EAC risk by about 40-fold [41,42]. During the last four decades, the incidence for EAC has dramatically increased [41,42]. Clinical studies provided well documented evidence that esophageal reflux of bile acids is also an important risk factor in the development of BE and EAC [42,43]. The exposure of esophageal epithelial cells to short, repeated episodes of bile acid reflux results in breaking of the epithelial barrier and stimulates cell proliferation. The rupture of cell membrane and cellular tight junctions in response to hydrogen ions and activated pepsin results in the mucosal and submucosal area of acid and pepsin penetration causing more severe hemorrhage and ulcerative damage. This combined action of acid and pepsin promotes esophageal epithelial differentiation to specialized intestinal-type glandular epithelium [44,45,46]. Besides gastric acid and pepsin, esophageal chronic reflux of bile acids and salts also predispose esophageal mucosa to development of BE and EAC [45], but the exact mechanisms underlying the transformation from specialized intestinal-type glandular epithelium to BE and EAC are still not fully understood and have become the subject of experimental and clinical investigations.

There are two isoforms of cyclooxygenases (COX) involved in the process of inflammation and tumorigenesis. The constitutive COX-1 is homeostatically expressed in most tissues while the inducible isoform COX-2 activated by numerous cytokines is detectable mostly in inflamed tissues [46,47]. The experimental and clinical evidence indicates the major role of COX-2 derived prostaglandins (PG) in the progression of specialized intestinal-type glandular epithelium to BE and EAC [48]. The process of carcinogenesis in animals and humans has been strongly linked to the COX-2 overexpression and the PG generation associated with decreased cell adhesion, increased cell proliferation and metastases formation, and the enhancement in angiogenesis, decreased apoptosis and increased immunosuppression [48,49,50,51,52]. The treatment with selective COX-2 inhibitors and NSAIDs has been shown to reduce the risk of EAC development, however, the therapeutic usefulness of these drugs is questionable because these COX-2 inhibitors were reported to exert local and systemic adverse effects [53]. According to recent consensus, BE is considered as an end-stage of GERD because this disorder is significantly more frequent among patients with RE than in the general population. Even though BE is commonly recognized as a condition preceding the development of adenocarcinoma of the gastroesophageal junction, the detailed mechanism of progression of RE into BE and esophageal oncogenesis remains largely unknown. It is widely accepted that clinically, BE involves at least 3 cm to fulfill the diagnostic criteria of so-called “classical” or “long segment” BE [54]. Currently, if the lesions are found in a segment shorter than 3 cm they belong to a “short segment” BE [54,55]. If the metaplasia is present and only manifest with irregularities or patches of properly localized Z-line, this particular case is referred as “ultra-short” BE [56]. Progression of Barrett’s metaplasia into EAC occurs as a result of chronic irritation of the mucosa by refluxate containing bile, since an apparent correlation between the bile salts concentration in refluxate and the degree of esophageal mucosal damage has been proposed [55,56,57]. The treatment of BE is mainly based on the chronic use of proton pump inhibitors (PPI) that reduce esophageal reflux acid gastric content and the surgical procedures aimed at restoring high pressure zones limiting the exposure of distal esophagus to irritating gastroduodenal refluxate [57,58]. Moreover, both methods are also used in combination with various endoscopic mucosal ablation techniques such as argon plasma beam coagulation (APC). All these procedures have been shown to enhance the process of re-epithelialization in the region of the GE junction [59]. There are no uniformly accepted standards of management of BE at present. It is proposed that the combined treatment of GERD with surgical ablation of metaplastic mucosa should minimize the risk of carcinogenesis. 

## 3. Role of Melatonin in Esophagoprotection and Prevention of Chronic Esophageal Disorders

**Figure 1:**
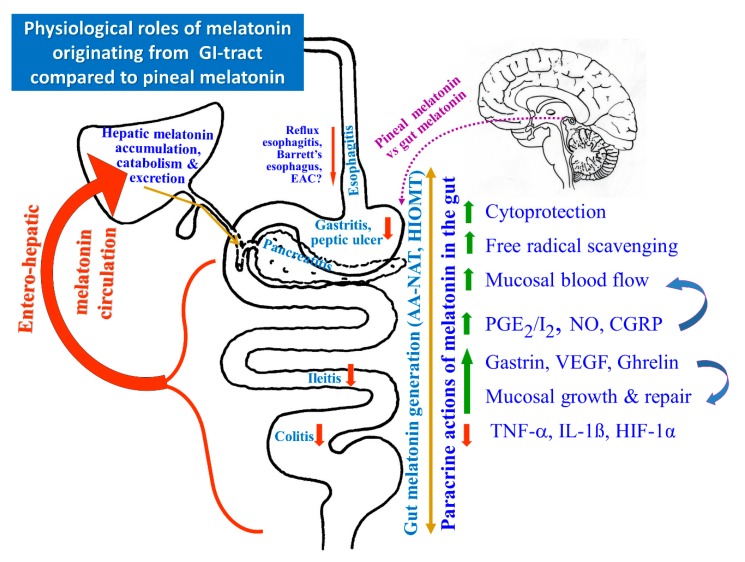
The mechanistic effects of endogenous melatonin produced in the pineal gland or gastrointestinal tract (GI-tract) and exogenous melatonin in attenuation of inflammatory reaction and protection of GI-organs including the esophagus, stomach and intestine.

Melatonin, an indole enzymatically derived from L-tryptophan, is the most versatile and ubiquitous hormonal molecule produced in all animal species and also in some plants [60]. Melatonin is synthesized from tryptophan in the mammalian pineal gland, however, a similar biosynthetic pathway of this indoleamine has been described in plants. In vertebrates, melatonin production is driven by the circadian rhythm with a peak at night [61]. Since melatonin is rapidly absorbed from the GI-tract the presence of this indoleamine and/or its precursor L-tryptophan in plant derived products is of particular importance. Fruits such as Citrus fresh and their juices are highly consumed worldwide. In *Citrus* genus plants, De Masi et al. [62] have reported that the labeled tryptophan is decarboxylated into tryptamine, the precursor of serotonin and melatonin. Furthermore, these investigators have revealed the existence of tryptophan decarboxylase (TDC) gene by in silico analysis [62]. In humans, melatonin is synthesized and secreted primarily by the pineal gland in response to environmental light/dark cycles, activated by suprachiasmatic nuclei (SCN), the major circadian oscillator, regulating the circadian rhythm of numerous biological functions. Previous studies revealed that the biosynthesis and plasma levels of this hormone are attenuated by age and various neurodegenerative, metabolic and cardiovascular diseases [63,64]. Melatonin acts on the target cells either directly or via G-protein coupled membrane receptors, including melatonin MT1R, MT2R and MT3R known to trigger several intracellular messengers such as cAMP, cGMP, and cytosolic Ca^2+^ concentration [65]. Similar to pineal gland cells (pinealocytes), the entero-endocrine (EE) cells present in GI-tract mucosa are the major source of intestinal melatonin [64,65]. After the termination of the biosynthesis process in EE cells, melatonin is immediately transported to the extracellular fluid, gastrointestinal lumen and its concentration increases in systemic circulation. From all these compartments it easily passes through all the membranes [66] (Figure 1) and can act on target cells in the GI-tract in both a paracrine and an endocrine manner when released into the portal vein [67]. Huether et al. [68] demonstrated that oral application of L-tryptophan causes a rapid elevation of circulating melatonin in rats. This rise in plasma melatonin levels was higher than that obtained after intraperitoneal administration of this indoleamine and this increment was abolished by portal vein ligation. Interestingly, this increase in plasma melatonin levels was unaffected by pinealectomy, indicating that the GI-tract is the major source of circulating melatonin after an oral application of L-tryptophan [68,69]. These studies also revealed that GI-tract mucosa contains melatonin under fasting conditions, however, its content in the GI-tract increases markedly after meal ingestion or consumption of a tryptophan-rich diet [69].

**Figure 2:**
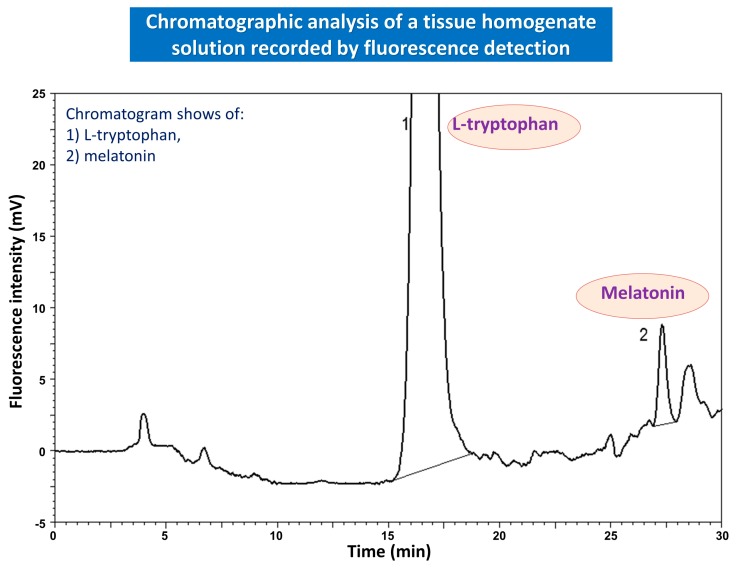
Chromatographic analysis of the tissue homogenate solution recorded by fluorescence detection presenting the conversion of L-tryptophan administered intragastrically in a dose of 100 mg/kg into melatonin in rat gastrointestinal tract. Chromatograms show particular peaks of melatonin derived from L-tryptophan in gastric mucosal tissue homogenates.

Several studies using immunohistochemistry and radioimmunoassay (RIA) techniques, validated by HPLC, have confirmed the presence of melatonin in the mucosa of the gut and showed that EE cells are the major source of melatonin in the GI-tract [64,70]. High concentrations of melatonin in both the gastric and duodenal mucosa and large amounts of it excreted into the bile in humans were confirmed by studies performed by Messner et al. [71]. Moreover, the plasma levels of melatonin in portal circulation greatly exceeds that found in peripheral blood at various points of time in the circadian period, but especially after food intake [66]. Therefore, it has been proposed that melatonin acts as the universal biological signal mediating the inter-organ cross-talk between the GI-tract organs and the liver [72,73,74] (Figure 1). Melatonin production and its release from the pineal gland remains under photoperiodic control, while its concentration in the gut depends mainly on food intake and could serve as an example of regulation through the negative feedback mechanisms [73]. The concentration of major enzymes involved in melatonin synthesis, namely hydroxyindole-*O*-methyltransferase (HIOMT) and *N*-acetyltransferase (NAT) as well as melatonin concentrations in GI-tract mucosa have been shown to exceed by 100–400 times those in the blood plasma levels. This increase in GI-tract melatonin synthesis is potentiated mainly after intake of protein meals containing L-tryptophan [74]. Thus, the enhanced production of melatonin in the GI-tract, as well as a L-tryptophan diet, contributes to the maintenance of this indoleamine concentration in peripheral blood [74,75,76] (Figure 2). Studies using a 2-[^125^I]-labeled melatonin revealed the binding sites for melatonin distributed in tissues of rodents and humans [68,74,75,76]. The binding sites for melatonin have been identified in all GI-tract tissues and the hyperbolic shape of specific binding curves confirmed that the radio-ligand is bound to a saturable number of binding sites possessing a single family [76]. Importantly, the tissue melatonin concentrations measured by RIA were about 20 times lower in the gut when compared to the pineal gland [77]. Interestingly, L-tryptophan administered orally raised melatonin not only in the pineal gland, but also in the GI-tract and the liver by about 6 and 10-fold, respectively [78]. L-tryptophan also increased the circulating levels of melatonin, mainly in the portal circulation. The local alterations in melatonin levels in the GI-tract following tryptophan application were unaffected by pinealectomy but significantly reduced by the partial occlusion of the portal vein [29,66,75,76,78].

**Figure 3:**
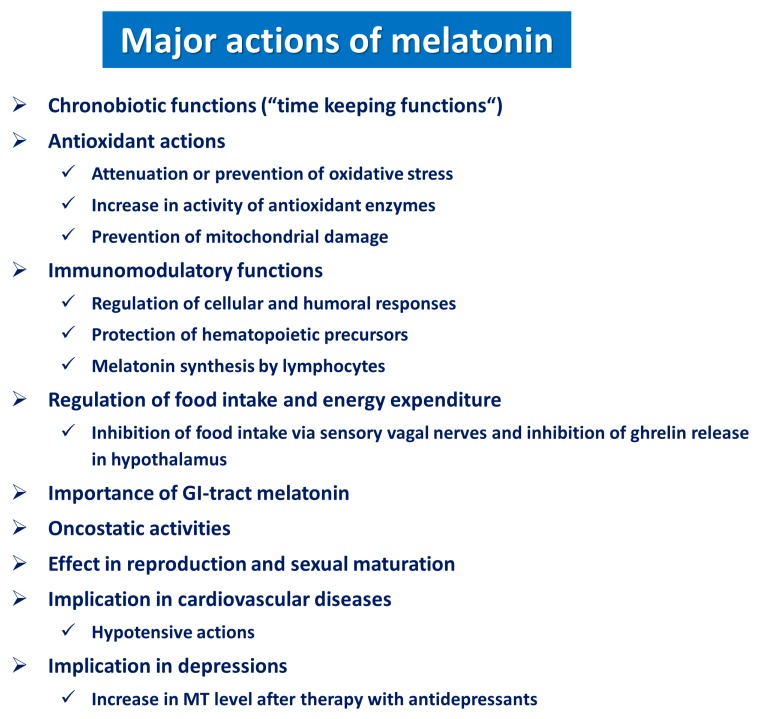
A summary of the pleiotropic actions of melatonin in gastrointestinal and extra-gastrointestinal organs. Besides regulating the circadian rhythm, melatonin plays an important role in the mechanism of protection of gastrointestinal organs including the esophagus, stomach and intestine.

These studies provided strong evidence that the GI-tract, particularly the stomach, duodenum and the hepatobiliary system, exhibits a high biosynthetic activity for melatonin, especially after tryptophan administration or high protein meal ingestion [29,78]. Large amounts of extra-pineal melatonin have been detected in tissues that are continuously exposed to a hostile environment, such as stress conditions affecting the GI-tract mucosa. The major function of locally produced melatonin in the GI-tract is to strengthen the esophageal and gastric mucosal barrier and to afford protection against the damage to esophageal and gastric mucosa against the deleterious action of various stressors including mucosal irritants of endogenous or exogenous origin, such as cytotoxins present in the digested food, alcohol consumption and NSAIDs ingestion [72,73,77,78]. The upper GI-tract, especially the esophagus is exposed to a variety of irritants entering the gut together with ingested food or local stressors including acid, pepsin and/or bile salts refluxed to the esophageal compartment with gastroduodenal content [29,78,79]. Nowadays, GERD without or with erosive changes in esophageal mucosa (non-erosive reflux disease (NERD) is often diagnosed as a condition that may lead to dangerous clinical complications, such as chronic esophagitis, esophageal ulcers, strictures, Barrett’s esophagus or Barrett’s adenocarcinoma [79,80]. Interestingly, Pereira et al. [79] reported that patients who suffered from GERD, showed remarkable remission of GERD symptoms following dietary supplementation of melatonin and L-tryptophan. The clinical remission of GERD symptoms and the number of patients’ complaints were comparable with those recorded in patients treated with PPI such as omeprazole [79]. Thus, melatonin or its precursor, L-tryptophan, accelerated the remission of GERD symptoms without any significant side effects, and that is why melatonin or L-tryptophan were postulated as possible alternative medications in GERD therapy [81,82]. Studies in rats revealed the presence of immunostaining for melatonin in the esophagus, particularly after administration of exogenous melatonin [75]. Marked and widespread esophageal lesions, including perforation of the esophageal walls, have been observed in experimental models of GERD in anesthetized rats when their esophagi were perfused daily for 2 h with acid-pepsin solution with or without addition of the bile [81]. Pretreatment with melatonin protected the esophageal mucosa against the formation of acute mucosal lesions, indicating the potent esophagoprotective activity of this hormone [81]. The activation of COX-PG and NOS-NO systems as well as the stimulation of capsaicin-sensitive afferent nerve endings by melatonin were implicated in the mechanism responsible for the protective action of this indoleamine [81]. Interestingly, pretreatment of rats either with indomethacin, the non-specific blocker of COX-1/COX-2, with N^G^ nitro-l-arginine (L-NNA), a non-specific suppressor of eNOS activity or with capsaicin, a neurotoxin known to induce e the functional ablation of the afferent sensory nerves, also abrogated the esophagoprotective effects of melatonin [81]. Moreover, indomethacin, used at a dose suppressing the esophageal mucosal generation of PGE_2_ by about 75%, or L-NNA which blunted the plasma nitrate/nitrite (NOx) level by about 60%, were more effective in suppressing the generation of mucosal PGE_2_ and plasma levels of NOx, respectively, in animals treated with melatonin than in vehicle-treated control rats [81]. The functional ablation of sensory nerves by capsaicin has been found to abolish melatonin-induced esophagoprotection [9,29,81]. Moreover, capsaicin deactivation of sensory nerves significantly attenuated the melatonin-induced increase in plasma NOx levels. Therefore, it was concluded that sensory nerves contribute to melatonin-induced esophagoprotection by releasing CGRP and NO due to NOS activation [29,81]. Previous studies using radiolabeled melatonin agonist 2-[^125^I] indolomelatonin documented the presence of melatonin binding sites in esophageal mucosa [75,76]. Therefore, it was proposed that the beneficial effects of exogenous melatonin could be attributed to its interaction with melatonin (MT) receptors [72,73]. The attenuation of melatonin-induced esophagoprotective action was linked with an impairment of mucosal blood flow caused by the pretreatment with COX or NO inhibitors such as indomethacin or L-NNA, respectively, and deactivation the sensory nerves by capsaicin. Thus, this evidence indicates that melatonin exerted its protective activity by increasing the EBF, possibly due to the synergistic activity and cooperative action of mucosal vasoactive mediators PG, NO and CGRP [81]. This notion is supported by the observation that pretreatment with indomethacin or LNNA abolished the esophagoprotective effect of melatonin that was accompanied by the fall in mucosal PGE_2_ generation and the amelioration of NO biosynthesis, respectively. These data indicate that PGE_2_ and NO were the major effectors responsible for protective activity of melatonin against acid-pepsin and bile perfusion in rats. Moreover, the capsaicin-induced deactivation of sensory nerves resulted in the most severe damage to esophageal mucosa exposed to the irritating action of acid and pepsin [83]. Since capsaicin suppresses the release of CGRP from sensory nerves that stimulates NOS expression, the activity of this enzyme and the NO biosynthesis, it is reasonable to assume that NO plays a central role in the mechanism of the esophagoprotection by this indoleamine. This is in keeping with the observation that reduced plasma levels of NOx in capsaicin denervated rats were reversed in part by co-treatment with melatonin or L-tryptophan [81]. In another experimental model of GERD, the effect of exogenous administration of melatonin and melatonin-derived endogenously from L-tryptophan with that of pantoprazole or ranitidine was studied in rats with RE evoked by two ligations, namely, (1) pylorus ligation and (2) the ligation of the limiting ridge between the forestomach and the corpus [29]. Four hours after the onset of gastric reflux induction, the mucosal lesions associated with edema of the submucosa and the fall in EBF was observed [29]. Histologically, the infiltration of numerous neutrophils has been noticed [29]. Pretreatment with melatonin or L-tryptophan or pretreatment with pantoprazole significantly reduced the lesion index (LI) and raised the EBF. Pinealectomy, which significantly decreased plasma levels of melatonin, augmented esophageal mucosa LI and these effects were attenuated in the pinealectomized animals pretreated with melatonin or L-tryptophan [29]. Luzindole, the MT2 receptor antagonist [65], abolished the melatonin-induced esophagoprotection and the rise in the EBF induced by treatment with this indoleamine. L-NNA or capsaicin, that by itself exacerbated esophageal injury and decreased EBF, also counteracted this esophagoprotection and the accompanying rise in EBF evoked by this indoleamine. Both these effects were restored with exogenous L-arginine and CGRP given in combination with melatonin. The increased expression of mRNAs for proinflammatory cytokines such as IL-1β and TNF-α and the rise in plasma IL-1β and adhesion molecules have been observed in experimental models of esophagitis [84], and these alterations in esophageal mucosa were significantly attenuated by pretreatment with melatonin and L-tryptophan [29]. These results have confirmed that melatonin-induced esophagoprotection against acid reflux-induced esophageal damage is mediated via the indoleamine activation of MT2 receptors, NO and CGRP released from sensory nerves and the downregulation of expression and release of proinflammatory cytokines TNF-α and IL-1β [29,71,82]. Melatonin has been recognized as a potent ROM scavenger and antioxidant capable of influencing all major physiological functions of the GI-tract including secretion, motility, digestion and intestinal absorption. Melatonin was reported as a beneficial molecule in scavenging the ROM. Besides exhibiting anti-oxidizing and anti-inflammatory actions, melatonin has also been shown to inhibit the formation of metalloproteinases-3 and -9, both strongly implicated in the pathogenesis of upper GI-tract injury and RE [78] (Figure 3). Under normal physiological conditions, the esophagoprotective activity of melatonin against GERD could be partly explained by its inhibitory influence on gastric acid secretion resulting in an increase in gastrin release [83]. Gastrin is known to increase the contractile activity of the lower esophageal sphincter (LES), thus reducing the incidence of gastro-esophageal reflux [83,84]. This may imply that melatonin exerts its beneficial gastro- and esophagoprotective actions also via melatonin receptors directly located on gastric G cells releasing gastrin, however, this hypothesis requires confirmation in further studies.

Although RE pathophysiology is multifactorial, transient LES relaxations may play an important role in the mechanism in esophageal dysfunction [85]. Frequently, patients with RE experience an increase in acid and activated pepsin exposure of esophageal mucosa compared to healthy control groups. However, there are also patients who do not experience an increase in the number of transient LES relaxations in comparison to controls [85]. In another study, the esophageal Het-1A monolayer barrier function in vitro was investigated by measuring transepithelial resistance (TER) and FITC-dextran paracellular permeation with or without acid treatment [86]. In a series of experiments in vitro utilizing the esophageal epithelial cells, the increased expression and activity of myosin light chain kinase (MLCK) and extracellular signal-regulated kinase (ERK) phosphorylation were observed, but upon acid treatment, the Het-1A monolayer permeability was increased [86]. When the Het-1A monolayer was pretreated with melatonin before the addition of acid to the culture, the permeability and the expression and phosphorylation of MLCK and ERK were markedly decreased [86]. It was concluded that melatonin may preserve the esophageal epithelial cell monolayer barrier functions in response to acid challenge by suppressing the transcription, translation and activity of MLCK through ERK1/2 signal transduction [86]. Thus, this study suggested that melatonin application in RE patients might be of potential clinical benefit in strengthening the integrity and permeability of surface epithelial esophageal cells [86]. This notion can be supported by the fact that older people are at a higher risk of complications from persistent RE due to the fall in melatonin production in elderly patients [87,88]. It is also of interest that patients presenting with upper digestive tract disorders such RE or duodenal ulcer show reduced plasma levels of melatonin [89]. This suggests that deficiency of this indoleamine may weaken esophageal and/or duodenal barrier mechanisms, thus exerting deleterious effects on the upper GI-tract mucosa [89]. This corroborates previous studies in humans with dietary supplementation containing melatonin and tryptophan, in which complete recovery from RE symptoms was observed after such treatment [89]. 

Melatonin is a natural compound and its safety and tolerability as a dietary supplement that mimics the function of endogenous melatonin in humans, has been well documented [87,88]. However, some adverse effects of the short-term or long-term melatonin administration should also be considered. For instance, the different administration times of this indoleamine, which is distributed in many countries over the counter, can influence the impact the compound has on circadian rhythm. For instance, the morning administration of melatonin can delay circadian rhythm and delay the onset of evening fatigue [89,90]. Melatonin was reported to improve sleep latency but the evidence for the overall improvement in sleep in humans is limited [91]. Still the potential concerns of daytime drowsiness increases when the administration of melatonin in daily hours is considered [92]. Residual daytime sedation, tiredness at rising, and increased sleep disruption with the use of melatonin have also been reported [93]. On the other hand, no convincing reports of local or systemic GI-tract adverse effects have been linked with chronic melatonin ingestion in humans, except for one incidence where it exacerbated the symptoms of the Crohn’s disease [94]. In several studies on melatonin safety, no serious adverse reactions with melatonin administered even in large pharmacological doses were reported [95].

Long term use of PPIs has been shown to be effective in GERD and BE patients [80,87,96]. These antisecretory drugs were shown to provide pain relief, prevent stricture formation and are relatively well tolerated and safe [83,88,96]. However, the common side effect of long term PPI administration is hypergastrinemia followed by the upregulation of COX-2 expression and an increase in PG activity observed in BE [88]. These effects could be mediated directly by gastrin acting via CCK_2_ receptor [97,98,99] or via an indirect pathway due to induction of EGF that in turn, could upregulate the expression of COX-2 due to EGF/TGFα receptor transactivation [97,100]. It is of interest that GERD can be a “physiological” process on a daily basis frequently observed in infants and that it becomes exacerbated by the structural and functional immaturity of the gastroesophageal junction, recumbent position and food consistency, including a liquid milk-based diet [101]. Further studies are still needed to prove whether acid suppressive drugs (PPI, i.e., pantoprazole) with or without selective COX-2 inhibitors (i.e., celecoxib) to inhibit the inducible COX-2 activity could be useful as therapeutic options in the treatment of BE in experimental animals and humans. 

## 4. Conclusions

As presented in this review, besides its pleiotropic activities in many systems including the cardiovascular, neurological, and endocrine systems (Figure 3), melatonin exerts a potent protective activity against endogenous (acid, pepsin) and exogenous (alcohol, NSAIDs, stress) damaging factors affecting the GI-tract. Melatonin has been shown to afford esophagoprotection against acute esophageal injury from acid and alkaline reflux in animals, but whether melatonin supplementation can protect patients with GERD from esophageal erosions and BE, and from developing neoplasia in humans, requires further clinical trials. This issue is of critical importance especially for older patients who are at higher risk of complications from persistent GERD, a disease which affects the cost-effectiveness of drug therapy, and further research should include strategies to reduce the financial cost of medical care and hospitalization in elderly.
